# Traumatic bone cyst of the mandible in Langer-Giedion syndrome: a case report

**DOI:** 10.1186/1752-1947-8-387

**Published:** 2014-11-25

**Authors:** Shakil Ahmed Nagori, Anson Jose, Bhaskar Agarwal, Krushna Bhatt, Ongkila Bhutia, Ajoy Roychoudhury

**Affiliations:** Department of Oral and Maxillofacial Surgery, CDER, All India Institute of Medical Sciences, Ansari Nagar, New Delhi, 110029 India

**Keywords:** Bone cyst, Diagnosis, Langer-Giedion syndrome, Mandible

## Abstract

**Introduction:**

Langer-Giedion syndrome (trichorhinophalangeal syndrome type II) is an extremely rare disorder characterized by dysmorphic facial features, multiple exostoses, mental retardation and digit deformities. We report the first case of any maxillofacial pathology in such a syndromic patient.

**Case presentation:**

A 22-year-old Indian woman with mild intellectual disability presented with malaligned teeth. Routine radiographic screening demonstrated a large multilocular lesion in her right mandible. She had peculiar features such as short stature, short limbs, brachydactyly, and dysmorphic facial characters, which prompted us to evaluate her further. After findings of multiple bony exostoses she was diagnosed with Langer-Giedion syndrome. On surgical exploration of her right mandibular lesion an empty cavity was found suggestive of traumatic bone cyst. The lesion healed completely after 1 year without loss of vitality of any teeth.

**Conclusions:**

Although diagnosis and management of any maxillofacial pathology can be challenging in syndromic patients, our report suggests a possible correlation between traumatic bone cyst and Langer-Giedion syndrome. Clinicians should routinely screen these patients for any undetected maxillofacial pathology. In future cases of this syndrome, one should consider the possibility of traumatic bone cyst which may not require aggressive surgical management.

## Introduction

Trichorhinophalangeal syndrome (TRPS) is a rare multisystem disorder first described by Giedion in 1966. It is caused by chromosome 8 deletions or microdeletion; it is characterized by abnormalities of the hair (tricho), nose (rhino) and digits (phalangeal). Three variants of this syndrome have been described: type I, type II and type III [1]. TRPS type I is characterized by sparse slow-growing hair, pear-shaped/bulbous nose, large protruding ears, short stature and cone-shaped epiphyses of the phalanges with clinodactyly/brachydactyly [2]. A variant of this syndrome, TRPS type II was described by Langer (1969) and Hall (1974) [3]. Also known as Langer-Giedion syndrome, TRPS type II has features of TRPS I with multiple exostoses and intellectual disability [4]. Type III is the rarest and extreme form with severe mental retardation and brachydactyly [1].

Reports describing oral features of TRPS are scarce. Also, no case of TRPS II with specific maxillofacial pathology has been reported. In this paper we describe our experience in the diagnosis and management of multilocular intraosseous lesion of the mandible in a patient with TRPS II.

## Case presentation

A 22-year-old Indian woman born to normal parents with no siblings reported to our hospital for management of malaligned teeth. She was unusually short in stature with mild intellectual disability and dysmorphic facial features. Her school performance was poor and she reportedly had hearing problems since childhood. Her hair was fine, sparse and fragile with slight receding hairline. Also seen were thick eyebrows, protruding ears, a pear-shaped nose with broad nasal bridge and columella. Her lateral profile was concave with a retruded maxilla and a prognathic mandible. On intraoral examination mild crowding was seen in relation to maxillary and mandibular anterior teeth. Oral hygiene was poor. Molar relationship was Angle’s Class III bilaterally.

**Figure 1:**
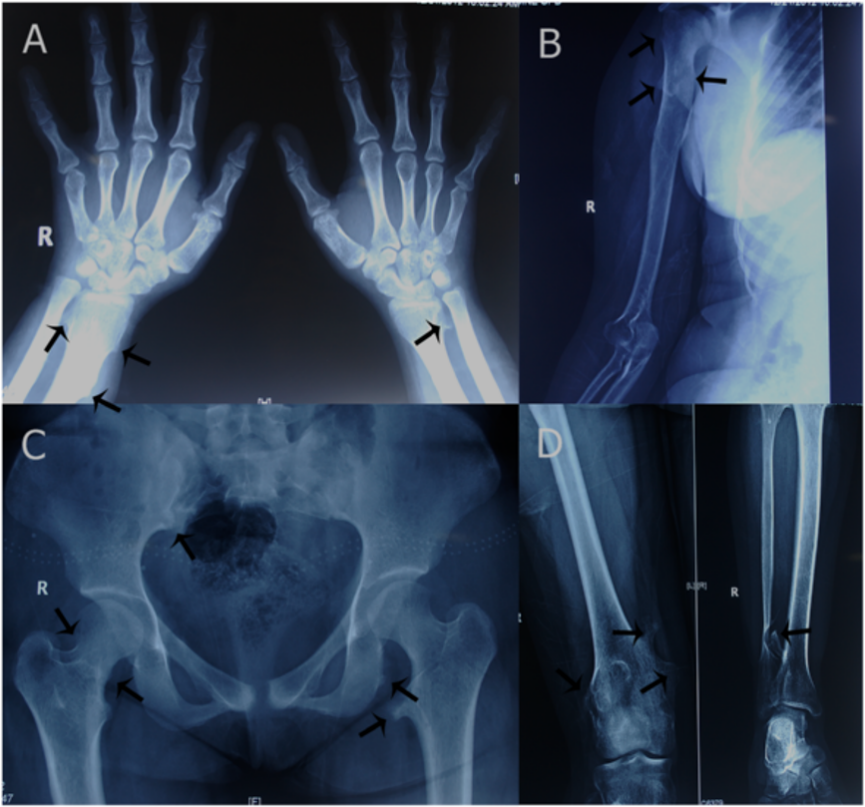
**Bony exostoses arising from multiple bones.** Exostoses (black arrows) seen on **A)** bilateral hand-wrist radiograph, **B)** right arm and shoulder radiograph, **C)** pelvis radiograph, and **D)** right knee and ankle radiograph.

She also had short limbs with brachydactyly of fingers and toes. Her wrist and interphalangeal joints were hyperextensible. A complete skeletal survey was carried out which demonstrated multiple bony exostoses in relation to bilateral ulna, right humerus, bilateral femoral, right tibia, right iliac and left pubic bone (Figure 1). There were no spine abnormalities. On hand-wrist radiographs, her bone age was estimated to be 19 years. Epiphyseal fusion had been completed so no cone-shaped epiphyses were seen. Her blood biochemistry was within normal limits. Her thyroid profile was normal. An echocardiogram revealed no cardiac abnormalities. However, ultrasonography of her abdomen and pelvis revealed infantile atrophic uterus and atrophic left ovary which had led to primary amenorrhea. Karyotyping demonstrated a normal female karyotype. Based on all these features a diagnosis of TRPS II was established.

**Figure 2:**
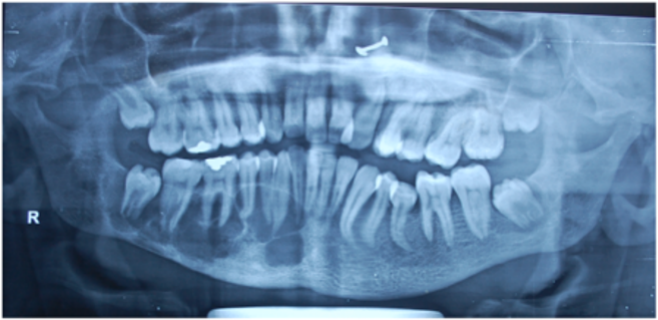
**Preoperative orthopantomogram showing a well-defined irregular multiloculated radiolucency in right half of mandible.** Note the developing third molars in all quadrants.

**Figure 3:**
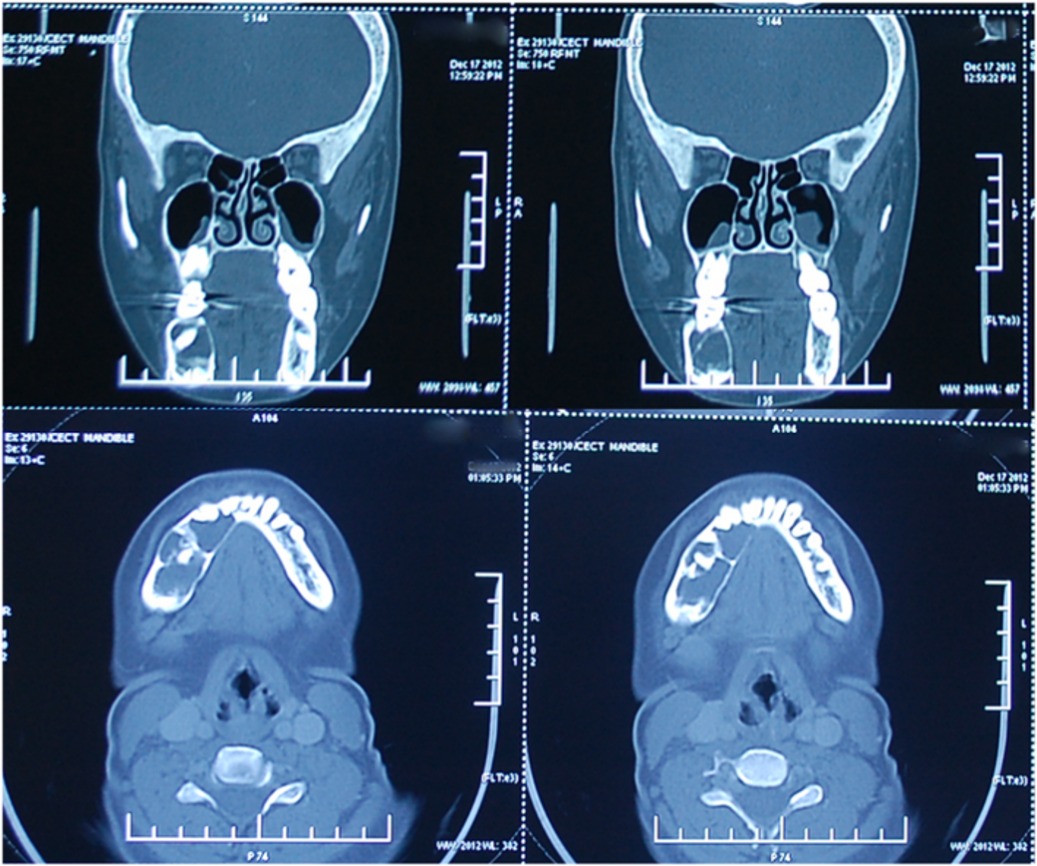
**Computed tomography scan showing a multilocular lytic lesion with mild expansion of buccolingual cortices and well-defined borders.**

An orthopantomogram carried out during the skeletal survey revealed a chance finding of a well-defined multilocular radiolucency in her right hemi-mandible extending from the midline to the angle region involving almost the entire height of bone (Figure 2). The lesion was seen extending interdentally but without any tooth displacement or root reabsorption. The inferior alveolar nerve canal was not traceable. On clinical examination, there was no paresthesia of her lower lip. Third molars were still in root formation stage. A computed tomography (CT) scan revealed mild buccolingual expansion of the right half of her mandible. A well-demarcated, multilocular lytic lesion was seen with no cortical perforation. No air-fluid levels or enhancing component was seen (Figure 3). A small amount of blood-tinged cystic fluid was aspirated from the lesion.

**Figure 4:**
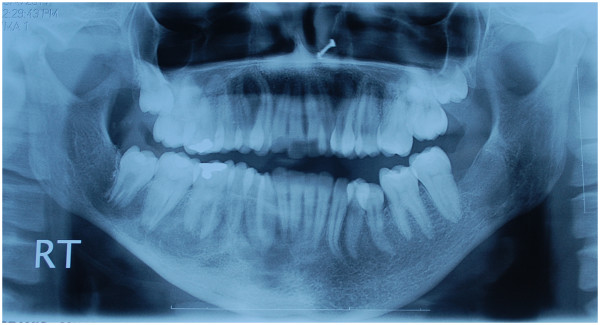
**Postoperative radiograph showing complete bone formation in the lesion at 1 year of follow-up.**

With a provisional diagnosis of a cystic mass, the lesion was explored under general anaesthesia via an intraoral approach. After creating a bony window beneath the tooth roots, an empty bone cavity with small amount of serosanguineous fluid was found without any epithelial lining. With features fitting with a diagnosis of traumatic bone cyst, bleeding was induced within the bone cavity and closure was done without extraction of any tooth. The radiolucency healed completely with good bone formation after 1 year of follow-up (Figure 4). There was no discolouration or loss of vitality of any tooth in her right hemi-mandible on follow-up.

## Discussion

The multiple abnormalities in patients with TRPS I are caused by deletions or heterozygous mutations in the *TRPSI* gene on chromosome 8q24.12. The more severe form TRPS II is known to occur due to a larger deletion at the same locus (from 8q24.11 to 8q24.13) [5] whereas TRPS III is caused by mutation of the same gene in the long arm of chromosome 8 (8q23.39) [2]. TRPS I and III are autosomal dominant conditions, TRPS II is usually sporadic. Diagnosis is based on clinical and radiographic features and genetic analysis may demonstrate a normal karyotype in some patients, as seen in our case [2, 6].

Typical facial features are seen in patients with TRPS II. They often have slow-growing, fine, sparse hair with high frontal hairline. Males may even have complete baldness by puberty. Eyebrows are thickened medially and may be thin or absent laterally. A bulbous pear-shaped nose is usually accompanied by thin upper lip and long philtrum. Ears are seen protruding and are described as "bat-like". The overall stature is short. Cone-shaped epiphysis especially of the middle phalanges is seen which leads to various amounts of brachydactyly [4]. Clinodactyly, that is radial angulation of the fifth finger, can be seen in some patients. The differentiating feature, however, is the presence of multiple exostoses which can sometimes cause pressure symptoms or aesthetic deformities [2]. Additional features that may be seen in TRPS II include: mental retardation, joint laxity, redundant skin and microcephaly [1]. Although almost all cases in the literature have been diagnosed in childhood, our patient was never examined by a clinician until 22 years of age. This was probably due to minimal functional problems caused by the syndrome and social stigma associated with syndromic patients in the Indian subcontinent. Schinzel *et al.*[4] have shown in their follow-up of four patients with TRPS II that typical cone-shaped epiphysis is usually seen up to puberty and such patients can lead near normal life up to adulthood. The absence of demonstration of cone-shaped epiphysis in our case is most probably related to late diagnosis.

Several nonspecific intraoral features of TRPS have been described in the literature namely malocclusion, crowding, multiple impacted teeth, microdontia, hypodontia, delayed tooth eruption, high caries index and maxillary/mandibular hypoplasia [1, 5, 7–9]. Supernumerary teeth have been frequently associated with patients with TRPS especially type I [10]. One report has also described underdeveloped condyle in a patient with TRPS I [7]. Our case demonstrated crowding, delayed development of third molars and maxillary hypoplasia with prognathic mandible. It was only after a complete skeletal survey did we find a lesion in her right hemi-mandible. Any unilocular/multilocular lytic lesion in the maxillofacial region in syndromic patients can be a diagnostic dilemma. Multiple supernumerary and impacted teeth can lead to formation of dentigerous cysts as in cleidocranial dysplasia and Gardner’s syndrome. Keratocystic odontogenic tumour is commonly seen in nevoid basal cell carcinoma syndrome [11]. Although such lesions are well-recognized features of these disorders, no such pathology has been associated with Langer-Giedion syndrome because it is extremely rare.

Considering the absence of symptoms, location at angle body region and minimal buccolingual expansion, keratocystic odontogenic tumour was a possible diagnosis in our patient. Also considered were solid ameloblastoma, central giant cell granuloma and aneurysmal bone cyst [12]. However, the absence of root reabsorption and displacement, nonenhancing lesion on CT scan, absence of ballooning and minimal blood-tinged fluid on aspiration complicated diagnosis. It was only after the intraoperative finding of an empty bone cavity that a diagnosis of traumatic bone cyst was established.

Traumatic bone cyst or solitary bone cyst is a nonepithelial-lined cavity, mainly seen in young individuals in the second decade of life with no gender predilection. It is more commonly located in the mandibular body between canine and third molar [13]. Asymptomatic in most cases it is usually discovered on routine radiographic examination. Associated teeth are usually vital with no reabsorption or displacement. It expands the cortices and, seldom, intraoral or extraoral swelling may be seen. On radiographic examination, a unilocular irregular but well-defined lytic lesion is seen characteristically extending between the roots of the teeth. Diagnosis is, however, invariably achieved after surgical exploration when the surgeon finds an empty cavity without any epithelial lining. In some cases small amount of fibrous tissue may be curetted from its walls. A few cases may contain some amount of blood-tinged fluid. The exact aetiology of traumatic bone cyst is not known. Trauma-haemorrhage theory has gained a lot of attention but has never been proved. It suggests that intraosseous hematoma formed after trauma to the jaws is reabsorbed by enzymatic process with destruction of adjacent bone [14]. The treatment of this condition is, however, universally accepted and involves surgical exploration and curettage to induce haemorrhage which subsequently forms bone [11]. These features were coherent with our case in which after inducing bleeding complete bone formation was visualized radiographically on follow-up. Although the management of traumatic bone cyst is quite conservative, it is important to note that remaining suspected lesions (keratocystic odontogenic tumour, ameloblastoma and aneurysmal bone cyst) require aggressive management [11]. Considering the rarity of TRPS II and lack of reports on specific dental findings, we cannot consistently relate the occurrence of traumatic bone cyst in these patients and our case may be sporadic in nature. However, it is important for clinicians to note the possibility of traumatic bone cyst in patients with TRPS II and resist initial aggressive management until proven otherwise.

An additional issue which may complicate management in such patients is their compromised intellectual level which makes thorough oral examination difficult. They are fearful and uncooperative (even our 22-year-old patient) and can have hearing difficulties [15]. For general anaesthesia, such patients may have difficult airways due to jaw deformities. Hypoplastic alae nasi may contraindicate nasal intubation, increasing the difficulty of intraoral surgeries. Joint hypermobility can manifest in the cervical spine with theoretical risk of luxation or subluxation with spinal cord injury. Spontaneous bone fractures are known to occur in such patients and one should be careful while positioning the patient. Skin laxity can also hinder intravenous access during surgery [15].

## Conclusions

Diagnosis and management of any maxillofacial pathology can be challenging in patients with TRPS. Our report suggests a possible correlation between traumatic bone cyst and Langer-Giedion syndrome. Clinicians should routinely screen these patients for any undetected maxillofacial pathology. In future cases of this syndrome, one should consider the possibility of traumatic bone cyst which may not require aggressive surgical management.

## Consent

As the patient is mentally incapable of making the decision, written informed consent was obtained from the legal guardians/parents of the patient for publication of this case report and any accompanying images. A copy of the written consent is available for review by the Editor-in-Chief of this journal.

## Abbreviations

CT: Computed tomography
TRPS: Trichorhinophalangeal syndrome.
